# Role of cardiac progenitor cell‐derived exosome‐mediated microRNA‐210 in cardiovascular disease

**DOI:** 10.1111/jcmm.14562

**Published:** 2019-09-26

**Authors:** Lirong Wang, Qiujin Jia, Chen Xinnong, Yingyu Xie, Yaqian Yang, Ao Zhang, Runteng Liu, Yuan Zhuo, Junping Zhang

**Affiliations:** ^1^ First Teaching Hospital of Tianjin University of Traditional Chinese Medicine Tianjin China; ^2^ Tianjin University of Traditional Chinese Medicine Tianjin China; ^3^ Department of Epidemiology, College of Global Public Health New York University New York NY USA

**Keywords:** biomarkers, cardiac progenitor cells, cardioprotection, cardiovascular disease, exosomes, MiRNA‐210

## Abstract

Cardiac progenitor cells are considered to be one of the most promising stem cells for heart regeneration and repair. The cardiac protective effect of CPCs is mainly achieved by reducing tissue damage and/or promoting tissue repair through a paracrine mechanism. Exosome is a factor that plays a major role in the paracrine effect of CPCs. By delivering microRNAs to target cells and regulating their functions, exosomes have shown significant beneficial effects in slowing down cardiac injury and promoting cardiac repair. Among them, miRNA‐210 is an important anoxic‐related miRNA derived from CPCs exosomes, which has great cardiac protective effect of inhibiting myocardial cell apoptosis, promoting angiogenesis and improving cardiac function. In addition, circulating miR‐210 may be a useful biomarker for the prediction or diagnosis of related cardiovascular diseases. In this review, we briefly reviewed the mechanism of miR‐210 derived from CPCs exosomes in cardiac protection in recent years.

## 1 INTRODUCTION

Cardiovascular disease (CVD) is still an important cause of human death, and its incidence is increasing year by year. Current treatments for these diseases slow the progression of the disease, but ultimately fail to prevent irreversible damage to the heart muscle, leading to pump failure. Therefore, how to repair and regenerate damaged cardiomyocytes is a key and difficult problem in the current treatment. Cardiac progenitor cells (CPCs) are a group of stem cells found in adult heart muscle and are one of the best stem cell types currently available to repair the heart. At first, it was believed that direct differentiation and replacement of damaged heart tissues after transplantation were the main mechanism of CPCs to promote cardiac recovery. However, with the progress of research, it was found that CPCs mainly reduced tissue damage and/or promoted tissue repair through paracrine. Exosome, an extracellular vesicle, is an important factor for heart repair and protection in the paracrine effect of CPCs. They are rich in important biological information such as proteins, lipids, mRNAs and MicroRNAs (miRNAs) and are important messengers and carriers for information transmission between cells and regulate the biological functions of other cells. Among them, miRNAs regulate cardiac functions by acting on themselves or distant target cells through autocrine or paracrine, so as to play a role in the treatment of CVDs. CPCs exosomes contain a variety of miRNAs, which have the effects of anti‐apoptosis, anti‐fibrosis, promoting angiogenesis, reducing infarction area and scar formation. Among them, miR‐210 is an important hypoxia‐related miRNA, which has certain advantages in inhibiting cell apoptosis, promoting angiogenesis and improving cardiac function. In this paper, the role and research prospect of CPC‐derived exosomes and miR‐210 in CVDs were reviewed in order to provide new ideas for the treatment of CVDs.

## 2 EXOSOME FROM CARDIAC PROGENITOR CELLS

Cardiac progenitor cells, also known as cardiac stem cells,^1^ are a small group of stem‐like cells in the heart, which are immature cardiac cells that can be differentiated into progenitor cells. Studies have reported that CPCs can promote myocardial cell proliferation, inhibit apoptosis, promote angiogenesis, reduce myocardial fibrosis and other beneficial effects and can increase myocardial blood flow, reduce inflammation, reduce the scar after myocardial infarction size, thereby increasing myocardial blood supply, reducing inflammation, reducing scar size after myocardial infarction, and even preventing myocardial injury after ischaemia/reperfusion, improving cardiac function.^2^, ^3^, ^4^ However, there has been considerable controversy about the main mechanism of CPC‐mediated cardiac repair, including the hypothesis of direct transdifferentiation and paracrine effect regulating endogenous repair.^5^ Early studies have shown that transplanted CPCs can be directly transformed into cardiomyocytes to repair or regenerate damaged hearts. However, in the process of a large number of experimental studies, it has been gradually found that CPCs mainly protects endogenous tissues through some indirect mechanism of action, thus promoting cardiac recovery. CADUCEUS clinical trial showed that cardiac function continued to increase after CPCs transplantation,^4^, ^6^ and the number of cardiac cells, blood vessels and endothelial cells increased after transplantation, all of which benefited from the paracrine effect of CPCs, but the indirect mechanism of the transplanted cells still could not be detected. Therefore, many studies have focused on the exploration of a paracrine mechanism of CPCs, and extracellular vesicles (EVs), as the basic medium of intercellular interaction, have attracted much attention.^7^ Researchers observed using transmission electron microscopy CPCs multivesicular body contains the secrete body sample EVs in mice and human heart,^8^ then studies further describe the ultrastructure of the outer body sample EVs secretion.^9^ The results showed that the diameter of EVs secreted by CPCs was about 30‐90nm and had a typical lipid double‐layer structure,^10^ which was similar in size and structure to exosomes. Therefore, they are exosomes and bioactive components that play a major role in the paracrine effect of CPCs.

The formation of exosomes begins with the process of invagination of cells, which form early nucleus (EEs). Under the control of related proteins, the early endosomes are formed by multiple intraluminal vesicles through the internal bud process. Polycystic bodies (MVBs) fuse with cell membranes under the regulation of Rab enzyme, secreting intraluminal vesicles, that is exosomes.^11^, ^12^, ^13^ Different types of cells secrete exosomes in normal or pathological conditions, carrying biologically active molecules such as proteins, lipids and nucleic acids, including mRNA, miRNA, long‐chain non‐coding RNA (lncRNA) and DNA. It has its specificity due to different sources. In vivo, secreted vesicles can be internalized by adjacent cells or circulated in the blood, eventually interacting with the cells within a certain distance, affecting the physiological pathways in the recipient cells.^14^ The means of information transmission between exosomes and target cells mainly include (a) ligands on the exosome membrane activate downstream signalling pathways; (b) activated receptors in the exosomes are transported between cells; (c) the proteins contained in the exosomes can be used for protein delivery; (d) the miRNA or RNA in the exosome content can be transferred to the genetic material. The membranous structure of exosomes can prevent the degradation of protein molecules carried by proteases in serum, and there are also specific cell binding sites on exosomal vesicles, which can fuse with cell membranes to make release specific proteins, lipids and RNA into the cytoplasm. After getting into recipient cells, exosomes can regulate the exchange of information between local and whole cells by transferring proteins, mRNAs and miRNA, etc, so as to induce corresponding physiological changes in the recipient cells. Therefore, exosomes can be used as the carrier of information transfer between cells in the body.

However, in many experiments, it has been observed that true transplanted cell regeneration is limited. In many cases, the number of neonatal cardiomyocytes and vascular cells after transplantation of CPCs is too small to explain improved cardiac function and morphology. Studies have shown that adult c‐kit (+) CPCs preferentially differentiate into endothelial cells and smooth muscle cells, rather than cardiomyocytes,^15^ and there are many obstacles in developing into mature cardiomyocytes in CPCs. The survival rate of CPCs is lower after transplantation in human and animal models, at the same time, undifferentiated cells also present a risk of developing teratomas.^16^ This series of questions has grown up to be key factor limiting the effectiveness of CPCs treatment. The secreted exosomes of CPCs contain special substances, which can be absorbed by the transplanted cells to improve their survival rate in the heart, avoid problems such as teratoma formation and limiting cell proliferation and differentiation during CPCs transplantation. Hypoxia can induce the release of exosomes from CPCs and change their molecular weight and activity, which enhances the migration of endothelial cells, increases capillary density, inhibits oxidative stress‐induced apoptosis, promotes cell proliferation and anti‐fibrotic effects.^10^, ^17^, ^18^ Chen et al found that exosomes secreted by mouse CPCs have anti‐apoptotic activity in the myocardial model of ischaemia/reperfusion (I/R) injury, which mainly inhibits the activation of caspase‐3/7 in the model of acute myocardial infarction (AMI) by the damage of H9C2 myocardial cells induced by H2O2, thus reducing the apoptosis of cells.^19^, ^20^ Experimental studies have shown that CPC exosome of mice has the effect of pro‐angiogenic and anti‐fibrotic.^18^, ^21^Among them, the pro‐angiogenic activity of CPC exosome in vitro and in vivo was mainly caused by the stimulation of endothelial cell migration by its matrix metalloproteinase (MMP) content.^22^ In different animal models of myocardial infarction (MI) (mice and pigs), injection of CPC‐derived exosomes at the edge of MI reduced infarct size, increased vascular density and recovered left ventricular ejection fraction compared with that of earlier studies.^10^, ^18^, ^23^

Exosomes derived from CPCs have the same cardioprotective functions as CPCs, but, which are more stable and easier to save than cells. Moreover, exosomes carry a large number of bioactive molecules, such as mRNA and miRNA, which are conducive to signal transmission between cells, and are the important material basis for their function. Many studies have suggested that EVs secreted by CPCs have cardioprotective effects, and its potential mechanisms involve miRNAs.

## 3 BIOLOGICAL CHARACTERISTICS OF EXOSOME‐DERIVED MIRNAS

A large number of miRNAs closely related to their structure and function on the contents of exosomes have turned out to be involved in a variety of cardiovascular pathophysiological processes. MiRNA is a kind of endogenous short‐chain, single‐stranded non‐coding RNA consisting of 22‐24 nucleotides, which can be bind to specific complementary sequences in mRNA, induce mRNA degradation, inhibit protein transcription or translation and regulate the expression of many genes. The regulation of gene expression of miRNAs has shown to be widespread in all types of cells.^24^ A stretch of miRNA fragments can be encoded into sequences such as exons, introns or non‐coding regions of the genetic gene.^25^ The production of miRNA mainly includes the following steps: (a) miRNA‐related gene sequences of some protein‐coding genes in the nucleus are transcribed to original miRNA (pri‐miRNA) under the action of RNA polymerase. (b) Pri‐miRNA is cut and folded into precursor miRNA(pre‐miRNA) of 70‐100 base length by endonuclease. (c) Pre‐miRNA is transported into the cytoplasm via guanosine triphosphate (GTP)‐dependent mode under the action of the output protein 5 and are further cut and modified under the action of the relevant ribonucleic acid polymerase. (d) The mature double‐stranded RNA is decomposed into two single strands of approximately 22 nucleotides in length, one of which is degraded as a passenger strand and the other becomes a guide strand into the exosomes.^26^, ^27^, ^28^ Some of the miRNAs in the circulation are combined with protein transport complexes. The other part is encapsulated in the circulating exosomes. The membrane structure of the exosomes enhances the ability to protect miRNAs, allowing circulating miRNAs to be stably present and not degraded by nucleases. By carrying and releasing such genetic material, exosomes achieve the purpose of transmitting information between functional RNAs, *that is*, miRNAs, between different cells.

Analysis of miRNA expression in exosomes derived from CPCs revealed that several overexpressed major miRNAs were enriched in CPC exosomes, including miR‐132,^10^, ^29^ miR‐210,^10^, ^21^ miR‐21,^30^, ^31^, ^32^ miR‐17,^21^ miR‐103,^21^ miR‐146a,^18^, ^33^ miR‐133a,^34^, ^35^, ^36^ miR‐451,^20^ miR‐20a,^21^ miR‐15b,^21^ miR‐181a and miR‐323‐5p,^10^, ^18^ whose biological effects are very broad, such as regulating cell differentiation and proliferation, angiogenesis, inhibition of apoptosis and fibrosis, playing an important role in cardiac protection (Table 1).

**Table 1:** Effects of major microRNAs (miRNAs) from cardiac progenitor cells (CPCs) exosomes on cardiovascular function

miRNAs from CPC‐ derived exosomes	Biological effect	References
miR‐132	Stimulate angiogenesis, inhibit cardiomyocyte apoptosis	10, 29
miR‐210	Promote angiogenesis, inhibit cardiomyocyte apoptosis, improve heart function	10, 21
miR‐21	Inhibit cardiomyocyte apoptosis, stimulate angiogenesis, promote endothelial cell proliferation	30, 31, 32
miR‐17/miR‐103	Promote angiogenesis, inhibit myocardial fibrosis	21
miR‐146a	Inhibit cardiomyocyte apoptosis and myocardial fibrosis	18, 33
miR‐133a	Inhibit cardiomyocyte apoptosis and myocardial fibrosis, improve cardiac function	34, 35, 36
miR‐451	Inhibit cardiomyocyte apoptosis	20
miR‐20a/miR‐15b	Stimulate angiogenesis	21
miR‐181a/miR‐323‐5p	Promote angiogenesis, cardioprotective	10, 18

## 4 CPCS EXOSOME‐MEDIATED ROLE OF MIR‐210 IN CARDIOPROTECTION

MiR‐210 is considered to be the most significant hypoxia‐related miRNA in the body, which is induced almost in all ischaemic diseases. Under hypoxic conditions, hypoxia‐inducible factor‐1α (HIF‐1α) is activated, allowing tissues to adapt to hypoxia. HIF‐1α can induce the expression of multiple miRNAs, which in turn can regulate HIF‐1α through a positive or negative feedback loop. Among them, the expression of miR‐210 was mainly induced by HIF‐1α, which binds to a specific site of the miR‐210 promoter to activate its expression. In anoxic myocardial cells, the expression of miR‐210 can also be up‐regulated by Akt, p53 and the upstream regulatory factor NF‐kappa B transcription factor p50 (NFkB1) of miR‐210, thereby reducing the production of mitochondrial ROS and exerting the protective effect on the myocardium.^37^, ^38^ Insulin can induce miR‐210 expression through the PI3K/Akt pathway.^39^ The study has also found that overexpression of miR‐210 is also regulated by oxidized low‐density lipoprotein (ox‐LDL).^40^ MiR‐210 exerts its corresponding biological effects by regulating the expression of target genes. In normal hypoxic environment, it can induce the up‐regulation of miR‐210 and regulate the occurrence and development of cardiovascular disease in many aspects.^41^ Its role is mainly in inhibiting cardiomyocyte apoptosis, promoting angiogenesis and improving cardiac function.

### 4.1 Inhibition of cardiomyocyte apoptosis

Cardiomyocyte apoptosis, also called programmed cell death, is related to the regulation of mitochondria and death receptors, which can be caused by cardiac injury. Cardiomyocytes are terminally differentiated cells with little potential to divide, so it is important to control the damage of cardiomyocytes. MiR‐210 has been shown to be involved in cardiomyocytes apoptosis. Studies have shown that miR‐210 is involved in cardiomyocyte apoptosis. In ischaemic cardiovascular disease, ischaemia and hypoxia are the key factors that cause cell injury, so it is important in order to restore blood supply as soon as possible. However, reperfusion therapy under certain conditions may cause different degrees of myocardial damage. After resuming reperfusion, the ultrastructure, function, metabolism and electrophysiological structure of the cells are further damaged. This clinical phenomenon has been one of the decisive factors affecting the prognosis and survival rate of patients. In this pathological process, a large amount of reactive oxygen species (ROS) is produced, which causes different degrees of oxidative stress, causing apoptosis and necrosis. As the disease develops, ventricular remodelling occurs, affecting cardiac function and eventually causing heart failure. Studies have reported that CPCs exosome‐mediated miR‐210 can be associated with the regulation of apoptosis through a variety of pathways. Overexpression of mir‐210 in cardiac myocytes can reduce ROS production, thereby inhibiting apoptosis under oxidative stress. These functions are mainly attributed to miR‐210's ability to regulate mitochondrial metabolism and reactive oxygen generation through an electron transport chain and inhibit pro‐apoptotic genes.^37^, ^42^

The transformation of cell energy metabolism plays an important role in the survival of cardiomyocytes under oxidative stress. MiR‐210 can participate in this process and play the role of inhibiting apoptosis by changing the metabolism of cardiomyocytes. ISCU and SIRT3 play an important role in regulating energy metabolism, and down‐regulating and inhibiting its expression can also promote the transformation of energy metabolism and reduce oxidative stress injury of cells. Studies have found that under the condition of oxidative stress injury of cardiomyocytes induced by H2O2, miR‐210 can inhibit the expression and activity of ISCU and SIRT3's mRNA and protein levels, thereby promoting the transformation of energy metabolism and indirectly inhibiting the apoptosis of cardiomyocytes.^43^ Meanwhile, GPD1‐L is also the target gene of mir‐210^44^, ^45^ and participates in cell energy metabolism. MiR‐210 can prevent electron transfer from NADH to the mitochondrial electron transport chain by targeting inhibition of GPD1‐L, thereby promoting the transformation of energy metabolism.^46^

E2F3 is a downstream target of miR‐210. E2F3 belongs to the activated subpopulation and can rapidly promote the transformation of resting cells from G1 to S phase and play an important role in cell proliferation and apoptosis.^47^ In the model of rat, primary cardiomyocyte oxygen‐glucose deprivation/reperfusion (OGD/R), miR‐210 can inhibit OGD/R‐induced apoptosis of myocardial cell, which may be caused by miR‐210 directly inhibiting the protein expression of its target gene E2F3, thereby playing a protective role in cardiomyocytes.^48^ Another direct target of miR‐210 is an apoptotic protein (Casp8ap2), and the up‐regulation of miR‐210 can inhibit its expression, thereby reducing myocardial cell apoptosis and participating in the protective effect of ischaemic preconditioning on bone marrow mesenchymal stem cells.^49^ In addition, studies have confirmed that the overexpression of miR‐210 can activate the c‐Met pathway and inhibit the apoptosis of mesenchymal stem cells (MSC) induced by oxidative stress, thus further realizing the application of MSC in the treatment of myocardial injury.^50^ Insulin protects H9C2 cells from apoptosis induced by oxidative stress, its key mechanism is that insulin can activate the PI3K/Akt pathway and induce the overexpression of miR‐210, thus inhibiting apoptosis.^39^

**Figure 1:**
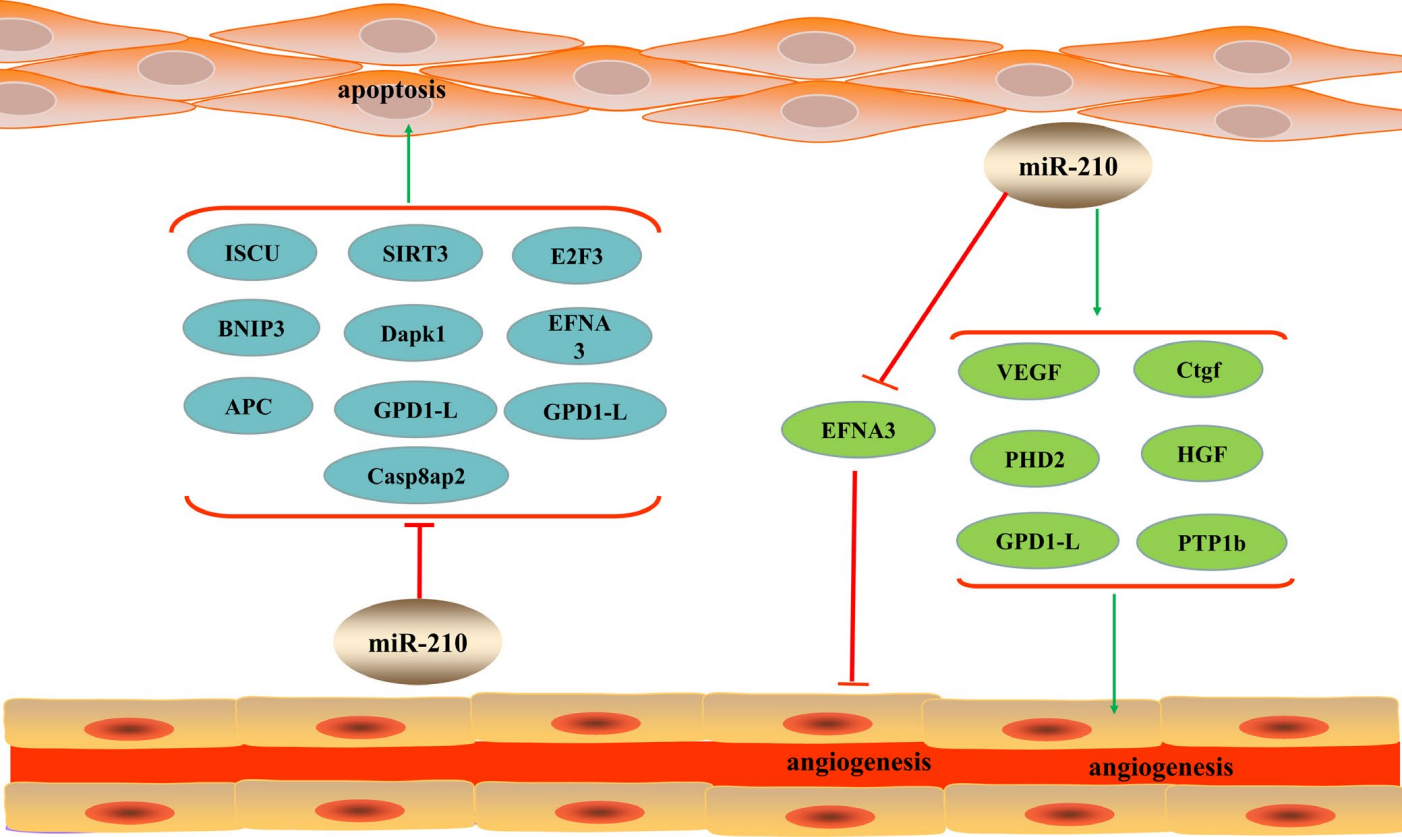
MiR‐210 plays the biological functions of inhibiting cardiac apoptosis and promoting angiogenesis by regulating the expression of related target genes. Green arrows indicate facilitation whereas red arrows indicate inhibitory

In addition, PTP1b, Ephrin‐A3 (EFNA3) and Dapk1 were also the target genes of miR‐210, and miR‐210 overexpression inhibited the apoptosis of myocardial cells after myocardial infarction by down‐regulating protein levels of PTP1b,Dapk1^42^ and EFNA3.^10^ APC is an anti‐proliferating gene and a direct target of miR‐210. Studies have confirmed that down‐regulation of APC mediated by miR‐210 overexpression can lead to proliferation of cardiomyocytes (CMs) and smooth muscle cells and reduce apoptosis.^51^, ^52^ The apoptotic protein BNIP3 plays important role in the process of apoptosis. It is target gene of miR‐210, which can inhibit myocardial apoptosis by down‐regulating the expression level of BNIP3 (Figure 1).^53^

### 4.2 Promoting angiogenesis

Acute myocardial infarction is prone to complications such as cardiogenic shock and chronic heart failure (CHF). The main reason is the decrease of myocardial blood flow brought about by coronary artery occlusion, which leads to myocardial cell death and myocardial remodelling. Therefore, improving myocardial function after AMI may play an active role in reducing the incidence of related complications and improving clinical outcomes. Early formation of collateral circulation may reduce the risk of myocardial cell death and myocardial remodelling after AMI, and angiogenesis can promote the ischaemic myocardium to grow new blood vessels with small blood supply capacity, establish an effective collateral circulation, thus recovering the blood supply of the ischaemic myocardium. It has been reported that the signal of angiogenic factor's increases in the cells expressed by miR‐210.^42^, ^54^ Therefore, it can be speculated that miR‐210 may also play a role through other effectors directly and indirectly. Vascular endothelial growth factor (VEGF) plays a critical role in the healing of ischaemic scar by regulating angiogenesis. As a transcription factor, HIF‐1 plays a role in angiogenesis by promoting VEGF gene transcription under hypoxic conditions, while miR‐210 plays a key role in this process. Overexpression of miR‐210 in endothelial cells stimulates capillary‐like structure formation and VEGF‐driven cell migration, while down‐regulation of miR‐210 inhibits this effect.^54^, ^55^ Arif et al^51^ injected miR‐210 into the myocardium of a rat model of acute myocardial infarction, observed angiogenesis in cardiomyocytes after four weeks and found that the vascular density in the area around the infarct increased by a factor of two, indicating that angiogenesis was enhanced; there was also a twofold increase in VEGF expression levels. This shows miR‐210 is directly concerned by the process of angiogenesis.

Hepatocyte growth factor (HGF) is an important stimulator in the process of angiogenesis, which can encourage the growth, proliferation and differentiation of endothelial cells (EC).^56^, ^57^, ^58^ Study has found that miR‐210 is overexpressed in acute myocardial infarction, and it can play a role in promoting angiogenesis of the myocardial infarction by targeting and regulating the up‐regulation of HGF, so as to improve new ventricular remodelling.^59^

EFNA3 is an important gene molecule and direct target of angiogenesis. Studies have found that overexpression of miR‐210 can play a role in promoting angiogenesis by lowering the level of EFNA3 protein in hypoxia,^60^, ^61^, ^62^ but some studies also believe that miR‐210 plays the above role by down‐regulating the level of EFNA3 mRNA.^42^ Meanwhile, Ctgf, PTP1b, GPD1‐L and PHD2 exert similar angiogenic effect under the regulation of miR‐210.^42^, ^63^ These studies proved that miR‐210 has a higher potential of angiogenesis, providing evidence for cardiac protective effect of miR‐210 (Figure 1).

### 4.3 Improve heart function

The continuous progress of various cardiovascular diseases will eventually lead to a decline in heart function, and eventually develop into an irreversible heart failure stage. Studies have found that miR‐210 exhibits a significant improvement in cardiac function in ischaemic heart disease. Hu et al^42^ showed that miRNA‐210 expression persisted for at least 8 weeks when plasmid carrying mir‐210 was injected into the myocardium of mice with AMI. The histological results showed that the myocardial infarction area was reduced, and the left ventricular systolic function was significantly improved by colour doppler ultrasound. Fan et al^59^ found that the cardiac contractility of rats with AMI receiving miR‐210 agonist was significantly enhanced, both LVEF and LVFS were improved. This result may be an effective explanation for the role of miRNA‐210 overexpression in microvessel formation. The main reason is that the blood flow is improved after the new blood vessels mature, forming a complete collateral circulation and increasing the supply of oxygen in the cardiomyocytes. The above experimental results are shown that both the reduction of infarct size and the increase of myocardial contractility are beneficial to the increase of the number of new blood vessels and the decrease of myocardial apoptosis. In summary, these good key features of miR‐210 can alleviate the pathological remodelling of scar tissue and regenerate new functional myocardium, thereby significantly restoring impaired cardiac function.

## 5 MIR‐210 AS A BIOMARKER OF CARDIOVASCULAR DISEASES

Because the membrane structure of exosomes enhances the ability to protect miRNAs, circulating miRNAs can stably exist in peripheral blood and resist degradation of endogenous RNases. And it can be detected in human body fluids. Therefore, some peripheral circulation miRNAs are considered as potential biomarkers of related cardiovascular disease.^64^, ^65^ In patients with aortic stenosis (AS), circulating miR‐210 significantly increased compared with healthy patients, and its expression level could predict the prognosis of patients.^66^ At the same time, miR‐210 is also auxiliary biomarkers and the prognosis of chronic heart failure. MiR‐210 under anoxic conditions present expression, and heart failure in pathophysiology, is caused by cardiac ejection in the body peripheral tissue relative lack of oxygen, so with the degree of heart failure, peripheral hypoxia also becomes more serious, miR‐210 expression levels are higher. The expression level of miR‐210 was significantly increased in patients with severe heart failure (NYHA III and IV), suggesting that miR‐210 could be used as a potential biomarker to predict the prognosis of patients with heart failure.^67^ However, research have shown that up‐regulation of miR‐210 can participate in the development of atherosclerosis by promoting endothelial cell apoptosis, and may become an effective therapeutic target for atherosclerosis,^68^ which is consistent with the miR‐210 over‐expressed in the serum of patients with atherosclerotic occlusion disorder, therefore, miR‐210 can be used as the biomarkers in the diagnosis of atherosclerosis.^69^ Therefore, we speculate that circulating miR‐210 may be a useful marker of predicting cardiovascular disease or diagnosis of related cardiovascular diseases and guide clinical treatment and prognosis.

## 6 FUTURE PERSPECTIVES

In recent years, stem cell therapy has become a research hot spot in the cardiovascular field, bringing hope to heart regeneration and repair, as well as questions and challenges. The potential of cardiac regeneration and repair of CPCs has been gradually valued. More and more studies have shown that the paracrine effect is the main mechanism of CPCs, which can activate the endogenous cardiac repair mechanism, thereby promoting endothelial angiogenesis, inhibiting myocardial apoptosis, anti‐fibrosis, reducing the scar after MI, and playing a role in cardiovascular protection, repair and regeneration. The exosomes derived from CPCs have the same cardioprotective function as CPCs and carry a large number of microRNAs that are closely related to their structure and function, which contribute to intercellular signal transmission. They participate in a variety of cardiovascular pathophysiological processes and are an important material basis for its functioning. MiR‐210 is an important hypoxia‐associated miRNA derived from exosomes of CPCs. Experimental researches have certificated that hypoxia conditions induce miR‐210 overexpression, which significantly inhibits cardiomyocyte apoptosis, promotes angiogenesis, improves cardiac function and embodies the heart of CPC protect and repair function. In addition, miR‐210 can enhance the role of mesenchymal stem cells (MSC) in the treatment of CVDs under hypoxia conditions, providing a useful therapeutic strategy for stem cell therapy in cardiac repair,^50^, ^70^ and miRNAs can be detected in many body fluids. Therefore, miR‐210 not only has unique advantages in the treatment of cardiovascular diseases, but also may be an excellent biomarker, which is expected to be an alternative to cell therapy. However, current research on miRNA still faces many problems. A miRNA can regulate multiple target genes, and multiple miRNAs can also jointly regulate the same gene. In addition, because the miRNAs and their potential targets are not fully complementary, it becomes very difficult to identify miRNA targets through informatic approaches. Therefore, it is impossible to accurately determine the mode of action of miRNAs and the mechanism by which it affects mRNA translation and gene expression. This complex regulatory network increases the limitations of clinical applications of miRNAs. At the same time, it is still not obvious whether circulating miRNAs is useful as meaningful biomarkers, and whether their detection methods are operable, and lack of large‐scale prospective studies to confirm. Therefore, the role of miR‐210 in cardiovascular disease has to be continuously explored and discovered in order to provide effective biological targets for clinical diagnosis and treatment.

## CONFLICTS OF INTEREST

The authors declare no conflicts of interest.

## AUTHORS' CONTRIBUTIONS

Lirong Wang is major contributor to the article, conceived, designed, wrote manuscripts, and drafted the figures and table; Qiujin Jia, Xinnong Chen, Yingyu Xie, Yaqian Yang, Ao Zhang, Runteng Liu, Zhuo Yuan, and Junping Zhang provided significant contribution to the revision of the manuscript.
